# Was the evolutionary road towards adaptive immunity paved with endothelium?

**DOI:** 10.1186/s13062-015-0079-0

**Published:** 2015-09-04

**Authors:** Gustav van Niekerk, Tanja Davis, Anna-Mart Engelbrecht

**Affiliations:** Department of Physiological Sciences, Stellenbosch University, Private Bag X1, Matieland, Stellenbosch, 7600 South Africa

**Keywords:** Adaptive immune system, Evolutionary constraint, Endothelium, Inflammation, Vertebrate, Invertebrate

## Abstract

**Background:**

The characterization of a completely novel adaptive immune system (AIS) in jawless vertebrates (hagfish and lampreys) presents an excellent opportunity for exploring similarities and differences in design principles. It also highlights a somewhat neglected question: Why did vertebrates, representing only 5 % of all animals, evolve a system as complex as an AIS *twice*, whereas invertebrates failed to do so? A number of theories have been presented in answer to this question. However, these theories either fail to explain why invertebrates would not similarly develop an AIS and are confounded by issues of causality, or have been challenged by more recent findings.

**Presentation of the hypothesis:**

Instead of identifying a selective pressure that would drive the development of an AIS, we hypothesise that invertebrates failed to develop an AIS because of the evolutionary constraints imposed by these animals’ physiological context. In particular, we argue that a number of vascular innovations in vertebrates allowed the effective implementation of an AIS. A lower blood volume allowed for a higher antibody titer (i.e., less ‘diluted’ antibody concentration), rendering these immune effectors more cost-effective. In addition, both a high circulatory velocity and the ability of endothelium to coordinate immune cell trafficking promote ‘epitope sampling’. Collectively, these innovations allowed the effective implementation of AIS in vertebrates.

**Testing the hypothesis:**

The hypothesis posits that a number of innovations to the vascular system provided the release from constraints which *allowed* the implementation of an AIS. However, this hypothesis would be refuted by phylogenetic analysis demonstrating that the AIS preceded these vascular innovations. The hypothesis also suggests that vascular performance would have an impact on the efficacy of an AIS, thus predicting a correlation between the vascular parameters of a species and its relative investment in AIS. The contribution of certain vascular innovations in augmenting immune functionality of an AIS can be tested by modelling the effect of different vascular parameters on AIS efficacy.

**Implications of the hypothesis:**

The hypothesis not only explains the immunological dimorphism between vertebrates and invertebrates but also brings to attention the fact that immunity is dependent on more than just an immune system.

**Reviewers:**

This article was reviewed by Dr. Jun Yu and Prof. Neil Greenspan.

## Background

The discovery of a novel form of adaptive immunity, based on variable lymphocyte receptors, has been considered “a total surprise” [1] and has been described as “arguably the most exciting finding of the past decade in immunology” [2]. This is hardly an over-exaggeration. As noted [3], comparative immunology has greatly expanded our understanding of the immune system by providing mechanistic insight into the functional intention underlying immunological structures. Furthermore, an understanding of the evolutionary context which gave rise to a structure as complex as the adaptive immune system (AIS) may provide novel insight into the underlying factors that drive evolutionary novelty [4, 5]. However, the evolution of two distinct forms of adaptive immune systems poses an interesting question in itself: Why did an AIS arise twice in vertebrates, corresponding to less than 1 % of all the animals that ever lived?

Theories attempting to explain the origin of an AIS often refer to chance events such as the two rounds of whole-genome duplication (2RoWGD) in vertebrates that would provide the genetic ‘raw material’ from which the AIS developed [1], or the accidental incorporation of a transposable element (bacterial [1] or viral [6]) that led to the development of the *RAG* genes (which play a critical role in generating the somatic variation necessary for an AIS). However, as recently contended [7], these approaches do not suffice in explaining the immunological dimorphism between vertebrates and invertebrates. As an example, *RAG* genes have been found in a number of invertebrates [8–10], yet these animals never evolved an AIS. Similarly, the 2RoWGD in vertebrates might have provided the genetic raw material for developing an AIS, but do not provide an answer as to how or why an AIS developed. In addition, the VLR-based AIS of jawless vertebrates (that do not make use of *RAG* genes), along with the demonstration that invertebrates are capable of somatic diversification without invoking an AIS [11], indicate that these ‘serendipitous’ events are neither necessary nor sufficient for developing an AIS.

Alternatively, theories have aimed to identify an evolutionary pressure that would ‘drive’ the development of an AIS. This approach is well exemplified by the intestinal biota hypothesis which proposes that an AIS developed as a means of cultivating complex symbiotic partnerships in vertebrates. There are a number of benefits associated with the expanded metabolic capacities made available by symbiotes [12] and evidence indicates that the AIS does indeed play a role in conditioning the composition of symbiote populations [13]. Yet it remains to be explained why invertebrates, that also make use of symbiotes [12, 14–16], would not similarly benefit from the expanded portfolio of intestinal biota and consequently evolve an AIS. In addition, a problem of causality arises: an AIS might have developed in response to pathogen stress and later acquired the additional role of screening symbiotic populations after the inception of an AIS.

## Presentation of the hypothesis

In all likelihood, a move towards a predatory lifestyle has promoted an increased metabolic turnover, and in turn, necessitated the development of a high-output vascular system, featuring a number of novel innovations [17–20]. One example is the notably low blood-to-body weight ratio seen in vertebrates, which is achieved by maintaining a high cardiac output coupled with high blood pressure [21]. Fish exhibit blood volumes ranging from 2–8 % of body volume [22, 23]. Compared to fish in general, lampreys have a high (~8 %) blood volume. Hagfish, however, exhibit the highest blood volume of all vertebrates (15–18 %) [24], which in part reflects the fact that these animals have among the lowest metabolic rates of all vertebrates [20, 25]. It should, however, also be noted that this high blood volume might have been a novel adaptation which is not reflective of original jawless vertebrates. Hagfish are habitually exposed to extreme anoxic conditions and exhibit a pronounced glycolytic capacity. In this regard, it has been remarked that the high blood volume (up to 30 % which is stored in large blood sinuses) may act as a metabolic buffer (e.g., to ‘dilute’ lactate build-up during anaerobic respiration [24]). Regardless, hagfish blood volume remains lower than most invertebrates [21, 26].

Blood volume may have critical implications for implementing an AIS since antibody binding to its target follows the law of mass action [27]: the amount of antibodies bound to epitopes is dependent on the equilibrium constant (i.e., the affinity between epitope and paratope) and the antibody concentration. Consequently, the low blood volume of vertebrates implies that higher antibody titer can be reached for an absolute amount of antibodies produced. Hence, a lower blood volume might have decreased the cost of utilising large globular immune effectors such as antibodies. However, cephalopods possess an exception to the general low-output vascular system of invertebrates, exhibiting a high-output cardiovascular system surpassing many vertebrates in terms of cardiac output and low blood volume [20] (for example *Octopus hongkongensis*, which has a blood volume of less than 6 % [26]). It is obvious that some overlap exists in terms of vascular output between the ‘high-range’ invertebrates and the ‘low-range’ vertebrates. Consequently, though lower blood volume may indeed play a role in increasing the efficacy of an antibody-mediated immune response, it does not present an unequivocal explanation for why an AIS developed exclusively in vertebrates.

Other vascular innovation includes the development of a closed circulatory system in vertebrates with bona fide endothelium [20, 28, 29]. Though certain invertebrates also possess endothelial-like cells, these cells lack critical futures such as cell-to-cell junctions and are therefore not truly endothelial [20, 28–33]. Indeed, these cells appear to be derived from blood-borne haemocytes (a type of immune cell of invertebrates) that are able to attach to the basement membrane of vessels and haemal cavities [29]. Though invertebrates do not possess true endothelium, the cells lining the vascular system of some invertebrates may perform similar functions, which include preventing turbulence, thus decreasing the cost of propelling blood along the vasculature [20].

**Fig. 1:**
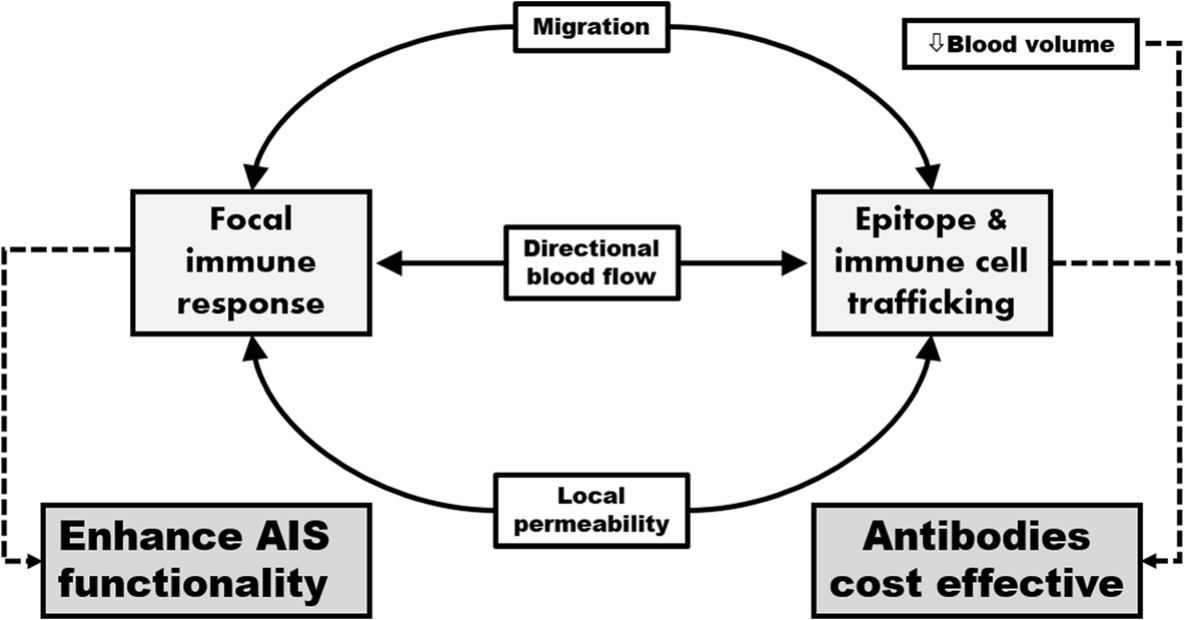
The vascular system plays a critical role in supporting the AIS. The lower blood volume of vertebrates allows for a higher concentration of antibodies thus facilitating binding kinetics and ultimately rendering antibodies cost-effective. Similarly, the endothelium controls local permeability of vasculature as well as the transcellular trafficking of immunoglobulins, thus facilitating a local increase in antibody concentrations. Moreover, endothelium cells promote directional blood flow and facilitate cell migration of B- and T-cells, thus exposing these cells to more epitopes which promote the efficacy of the AIS

Notably, in vertebrates, endothelium also controls the blood flow and the permeability of local vessels. These cells integrate multiple signals, and are particularly responsive to signals produced by immune cells [34, 35], signifying the close functional relationship between the immune system and vasculature. Other immunological functions include the recruitment of immune cells (e.g., secretion of chemotactic agents and promotion of macrophage ‘rolling’ over endothelium), presentation of antigens, transcytosis of antibodies or controlling vascular permeability (e.g., disengaging tight junctions) and, during prolonged infection, angiogenesis as well as the formation of tertiary lymphoid angiogenesis [34–38]. Collectively, these vascular innovations provide the logistical support indispensable in implementing an AIS-mediated immune response (Fig. 1).

**Fig. 2:**
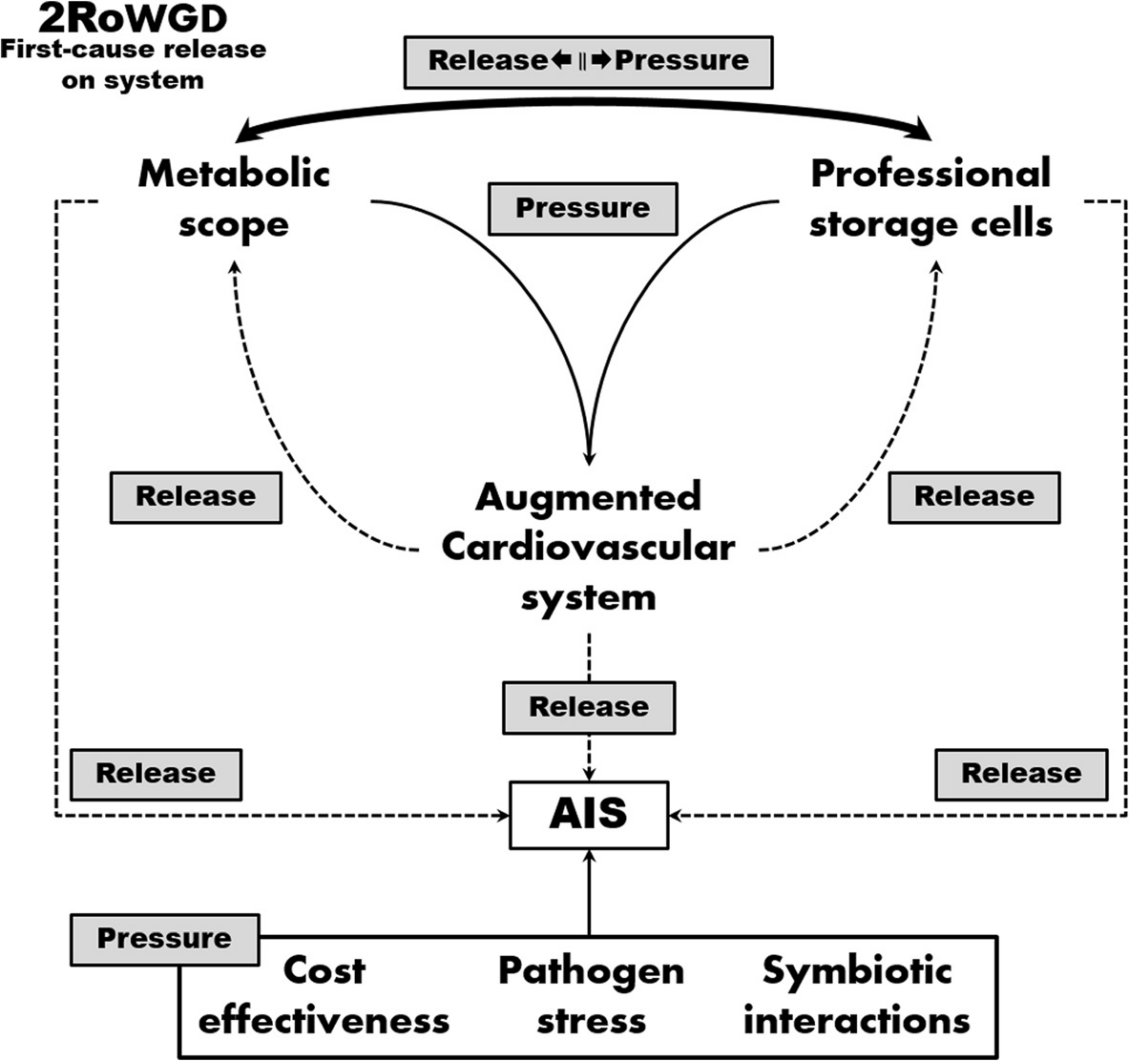
Schematic illustration of evolutionary forces designated as ‘pressure’ or ‘release’. Whereas previous theories have attempted to identify a selective pressure that would ‘drive’ the evolution of the AIS, we argue that the physiological context of vertebrates provided the release from evolutionary constraint that allowed the effective implementation of an AIS. A shift towards a more active lifestyle imposed the need for a higher metabolic scope. This includes the development of specialised cells for the storage of energy-rich molecules (adipocytes – an innovation unique to vertebrates [7]) to buffer against sudden supply or demand shocks and a high-output vascular system typical of vertebrates. The higher metabolic scope would allow for the implementation of a costly AIS, whereas vascular parameters decrease the total cost of ownership of an AIS and promote AIS functionality

In the context of our previous efforts [7], we propose a novel narrative to explain the evolutionary origin of an AIS (Fig. 2). A more active lifestyle necessitated an expanded metabolic scope which in turn resulted in the development of certain physiological innovations such as a closed vascular system and adipocytes. These innovations provided the evolutionary release from constraints that allowed the implementation of an AIS: whereas adipocytes buffer against the sudden metabolic demand associated with the activation of an AIS, vascular innovations decreased the cost of mobilising an antibody-based immune response and augmented the performance of an AIS (e.g., promoting affinity maturation).

## Testing the hypothesis

An extensive database for various vascular parameters would be required to inspect the effect of these vascular parameters (e.g., circulatory turnover and blood volume) on the implementation of AIS. Also, since it is argued that the closed vascular system provided the release from constraint, a number of vascular innovations must have preceded the advent of an AIS. If, for example, phylogenetic studies indicate that AIS developed before critical vascular innovations, it would strongly suggest that an AIS is not dependent on the vascular system in the manner argued here. However, since both the AIS and endothelium developed concomitantly for 500 million years, certain innovations to endothelium might have arisen only after the AIS was established (e.g., translocation of antibodies by endothelium mediated by Fc-receptor [38] evolved after immunoglobulins were implemented).

The hypothesis also predicts differences in an animal’s relative investment in an AIS. As an example, fish endothelium is generally not effective in preventing ultra-filtration [39]. This would suggest that fish might be less adept at utilising antibodies, since antibodies would potentially filtrate indiscriminately, thus requiring larger numbers to reach an effective concentration. Indeed, zebrafish lacking the *Rag*-1 gene (thus lacking an AIS) remain viable in non-sterile conditions [40], suggesting a lower dependency on the AIS. Comparing vascular parameters with immunological investment in an AIS may illustrate prevailing trends that support or refute this hypothesis.

## Implication of hypothesis

In addition to providing a novel explanation to one of the most fundamental questions in evolutionary immunology, and highlighting the critical role that evolutionary constraint might play in explaining the origin of evolutionary novelty, the hypothesis also emphasises the fact that immunity manifests as a system-level phenomenon. The functional integration between immune and non-immune systems in mediating immunity may lead to therapies targeting non-immune actors in order to manage infections. It may also explain forms of immunodeficiency.

## Reviewers’ reports

### Referee 1, Dr. Jun Yu

The authors asked an interesting question here but the answer (s) may not be as simple as a physiological one, especially the current beliefs or evolutionary hypotheses have not built enough evidence or arguments for such profound questions. For instance, did both vertebrates and invertebrates evolve from the same bilaterally-symmetric multicellular ancestral organism? Do we know the differences between invertebrate and vertebrate genomes? Let us stratify the question further and evaluate the current approach. First, the authors introduce the manuscript with the question: Why did vertebrates, representing only 5 % of all animals, evolve a system as complex as an AIS twice, whereas invertebrates failed to do so? The answer to this has to be addressed by comparing the genomic histories (genotypes) and physiological contexts (phenotypes) between vertebrates and invertebrates. The subjects are too big to be discussed here, but one thing we do know now is the fact that the genomes and their organizational principles of the two lineages are very different. For instance, the invertebrate genomes, especially arthropod genomes (as so far sequenced) can not be duplicated genome-wide, i.e., whole-genome duplication (WGD) does not exist in invertebrates, even though their individual genes have been duplicated enormously as needed. WGD has been known to produce the multiple HOX gene clusters in vertebrate genomes. Another instance is the complexity of epigenetic mechanisms that are biased toward RNA-based regulation in invertebrates and DNA-based regulation in vertebrates. Second, many new vascular inventions and anatomic features are certainly unique in vertebrates. The heart, for instance, has been evolved from a tube-like to a four-chambered structure in mammals. In a sharp contrast, invertebrates seldom have novel inventions as fossil records have shown little fundamental differences between modern insects and their counterparts found some 500 million years ago. Third, I suspect that without a detailed list of genes unique to the two lineages or any unique features one will not able to build any solid argument for new physiological inventions. To invent any complex cellular mechanisms must satisfy at least two requirements: involvement of multiple genes or gene families and origination from existing primitive mechanisms. Any physiological inventions must have origins of cellular mechanisms. After all, instead of making an apple-to-orange comparison, it may be better to compare inventions with a single lineage, such as within the vertebrates, as how does AIS evolve in the two rounds of vertebrate WGD.

Author response: One of the key aspects of the presented hypothesis is an appreciation of immunology as a physiological phenomenon. That is, the immune system is imbedded within a range of tissue systems. As such, we approach the question regarding the evolutionary origin of AIS with the view that “executing the immunological mandate requires more than just an immune system” [7]. However, it is also true that physiological innovations must ultimately relate to the effect of evolutionary forces acting on genomes. In this regard, we believe that understanding the physiological context in which a novel innovation arises will point to the relevant genes that might have driven the physiological phenomena that allowed the development of AIS.

As pointed out by the reviewer, 2RoWGD need not be the only source for novel gene functions. A number of genes in invertebrates have undergone repeated rounds of duplication, indicating that gene duplication may provide an alternative mechanism for generating novel functionality. Indeed, duplication of genomic regions in the kbp to Mbp range are an order of magnitude more frequent than point mutations in lower organism [41], suggesting that gene duplication may be a rich source of genomic ‘raw material’ from which novel gene function may evolve. Similarly, evolution in gene regulatory systems (as opposed to novel gene products) can also drive evolutionary novelty [4, 5]. Furthermore, genome duplication is not a guarantee for the generation of evolutionary novelties. Bony fish have undergone 3RoWGD (and trout 4RoWGD), yet these animals do not exhibit profound physiological or immunological complexities. Indeed, fish have very poorly defined secondary lymphoid tissue [42], indicating that WGD does not ‘drive’ immunological innovation. We speculate that mammalian physiology is better suited for implementing an AIS and as such these animals have invested more in developing supportive structures (such as well-developed secondary lymphoid tissue that plays a critical role in epitope sampling). In any case, these observations suggest that genome duplication is not a *sine qua non* for the development of evolutionary novelty. Thus, it is tempting to speculate whether other factors such as the ‘regulatory biases’ between vertebrates and invertebrate might play a more restrictive or permissive role in the development of evolutionary novelties.

However, the recently sequenced lamprey genome indicates that it is likely that 2RoWGD occurred before the split between jawed and jawless vertebrates [43]. It is a rather doubtful coincidence that two forms of AIS evolved in the only animals that also underwent genome duplication. As argued before [7], 2RoWGD might have played a key role in the development of neural crest cells of both jawed- [18, 44] and jawless vertebrates (lamprey [45] and hagfish [46]), which in turn resulted in physiological novelties (such as a sophisticated vascular system) that allowed the implementation of AIS. Thus, a physiological perspective may supplement a molecular approach: whereas “[g]enome duplication merely enhances the diversification potential of a lineage” [47], the physiological view describes *how* and *why* 2RoWGD could lead to an AIS (and why only in vertebrates).

As the reviewer has rightly pointed out, a major task now would be to catalogue the step-wise genomic events that allowed the development of AIS. Understanding how evolutionary novelty arises may also hold great clinical relevance, particularly in understanding how cancer cells develop novel phenotypes.

### Referee 2, Prof. Neil Greenspan

Van Niekerk et al. address the interesting evolutionary question. Why did complex adaptive immune systems (AIS) exploiting highly diverse antigen-specific receptors including secreted forms (i.e., antibodies) evolve in for vertebrates with jaws but not in invertebrates, which represent the overwhelming majority of animal species. Their provocative hypothesis suggests that a key factor accounting for the difference between vertebrates and invertebrates with respect to the presence of AIS may have been the physiological innovations found in vertebrates relating to circulatory systems. Specifically, the authors cite the relatively low blood volumes (relative to body mass) for vertebrates, the fact that vertebrates have closed vascular circuits, and the abilities of endothelial cells to regulate blood flow and cellular access to the parenchyma tissues. Given the hematopoietic origins of the cells most closely identified with immune-mediated phenomena and the major dependence of these cells and the functions they serve on transport by the circulatory system, the author’s hypothesis is plausible and worthy of further conceptual, theoretical, and experimental exploration. The authors offer a couple of testable predictions of the present hypothesis. They argue, for instance, that a key role of vascular physiology in the evolution of the AIS would be brought into question if the AIS could be shown to have evolved in some species before the vascular adaptations being cited as critical to AIS evolution. So, the authors’ proposal has a reasonable likelihood of fulfilling the purpose of a Biology Direct Hypothesis: in this case, promoting additional thinking about the evolutionary origins of AIS in vertebrates and stimulating new experimental studies relevant to this question.

Minor issues: p. 2, in “Testing the hypothesis” section, 3rd line from top - “ASI” should be “AIS”, p. 4 – “titter” should “titer” p. 9 – “include” should be “includes” p. 9 – “The higher metabolic scope allow” should be “The higher metabolic scope would allow” p. 10 – “promoted” should be “promote” Quality of written English: Needs some language corrections before being published.

Author response: We have corrected these errors. The manuscript has now also been language edited.

## Abbreviations

AIS: Adaptive immune system
2RoWGD: Two rounds of whole-genome duplication
